# The Influence of Film and Storage on the Phenolic and Antioxidant Properties of Red Raspberries (*Rubus idaeus* L.) cv. Erika

**DOI:** 10.3390/antiox8080254

**Published:** 2019-07-30

**Authors:** Angelo Maria Giuffrè, Lamia Louadj, Paola Rizzo, Emanuela De Salvo, Vincenzo Sicari

**Affiliations:** 1 Dipartimento AGRARIA—Università degli Studi “Mediterranea” di Reggio Calabria, Contrada Melissari, 89124 Reggio Calabria, Italy; 2 Dipartimento di Chimica e Biologia, Università degli Studi di Salerno, via Giovanni Paolo II 132, 84084 Fisciano (SA), Italy

**Keywords:** nanoactive film, PET, packaging, post-harvest, shelf-life, raspberry, rubus, small fruit

## Abstract

In this paper, the effect of the packaging material and storage method on red raspberries produced at Reggio Calabria (Italy) was studied. For this purpose, the fruits were stored immediately after harvest in different conditions: in the fridge at 1 °C and in the freezer at −20 °C, using different packaging materials, two new patented films (nanoactive A) and (nanoactive B), one common packaging film (polyethylene terephthalate), and other fruits were left without any packaging material. Chemical parameters were analysed at harvest to have the initial characteristics without any conditioned storage and to distinguish the post-harvest effect on the fruits, then daily for storage in the fridge and monthly for storage in the freezer. The aims of our project were first to determine the qualitative characterization of these red raspberries, the optimization of their shelf-life during time in the fridge or freezer, using the different types of packaging materials and finally to highlight the usefulness of the new patented packaging materials. Nanoactive A film showed the best shelf-life in the fridge, and after 14 days the values, given as mg/100 g fresh weight, were: total phenolics (166.70), monomeric anthocyanin content (50.82), flavonoids (24.64), ascorbic acid (32.42), and 1, 1-diphenyl-2-picrylhydrazyl (DPPH) assay (95.93).

## 1. Introduction

Over the last decade, the awareness of consumers about how food can positively affect their health, has been growing tremendously [1,2]. The European Commission and the World Health Organisation have recommended the consumption of 400 g (at least) of fresh fruit and vegetables in 4–6 servings per day to promote health by reducing the risk of some types of cancer and arteriosclerosis [3]. Consumers worldwide have become aware of the action of free radicals and antioxidants, and the antioxidant effects and composition of different fruits and vegetables have been the subject of much research. Oxidative damage of cellular and functional molecules, such as DNA, lipids, and proteins, is caused by Reactive Oxygen Species (ROS), such as superoxide, hydrogen peroxide, hydroxyl radicals, and free radical-meditated reactions [4,5]. This cell dysfunctional effect or oxidative stress was proved to be the evident major cause of many diseases including cancer, Alzheimer’s disease, cardiovascular disease, diabetes, and other neurodegenerative disorders, as well as aging [6,7]. The remedy to the ROS-mediated tissue impairments was managed effectively by antioxidants. Many antioxidant compounds with different efficiency levels have antitumor, anticarcinogenic, anti-inflammatory, antiatherosclerotic, antimutagenic, antibacterial, antiproliferative, or antiviral activities [8,9]. Consumer interest has focused on functional foods and nutraceuticals, naturally derived antioxidants, and their efficacy. Because of their significant antioxidant effect, berries have been increasingly consumed over the past two decades, especially in North America and Europe. Among these, raspberries are a good source of antioxidants (anthocyanins, flavonoids, Vitamin C, and phenolic acids) [10]. Red raspberries (*Rubus idaeus* L.) are consumed in different ways: fresh, frozen, dried, puréed, and as a jam or juice. These fruits have a short shelf-life of two days if consumed fresh and without refrigeration. In addition to their taste, berries possess benefits to human health, by their richness in micronutrients and phytochemicals such as anthocyanins, phenolics, and ascorbic acids. Some in vitro studies in berries, indicate that potential anti-heart disease and anti-cancer properties including antioxidant, anti-inflammatory, and cell regulatory effects are due to anthocyanins and other polyphenols [11]. Wang et al [12] found that the total phenolic content follows different trends during the ripening of the berries: the content decreased from the green to the pink stage, then subsequently, there is a significant increase in total phenolic content from the pink stage to the ripe stage. These stages of ripeness, together with fruit damage, influence the berries’ firmness and juice content. These organoleptic factors are important to consider during post-harvest handling, transportation, storage, and processing [13]. There is an increasing interest in packaging technology to improve the shelf-life of fruits and vegetables [14]. 

Packaging contains and protects food, moreover it informs the consumer [15,16,17,18]. Packaging makes the managing of fruit easier, in particular for small fruit or pre-portioned fruit. Fruit has to be protected from insects, mould, and environmental pollution by packaging, which also preserves the physicochemical properties of the fruit for as long as possible. In addition, packaging informs the consumer with data listed on the label (ingredient list, origin of ingredients, manufacturing, instructions for use, expiration date) and with that information, the consumer can visually evaluate the food through the package should this be transparent (color) or tactilely appreciate if it is flexible (consistence). Packaging, besides being a marketing tool, plays a key role in preserving food quality and safety. Polyethylene terephthalate (PET) is one of the most widely used films for packaging of foods. It is flexible, has extremely low electrical-resistance, has a good transparency, and an extremely high transparent electromagnetic interference shielding [19], minimizing the heat conduction during laser irradiation [20], and it is easy to find.

Active packaging includes oxygen scavengers, ethylene scavengers, flavour and odour absorber/releaser, and antimicrobials [21,22] into packaging systems, with the aim of extending food products quality and shelf-life.

The ability to prolong shelf-life of fruit and vegetables, by active films with a nanoporous crystalline syndiotactic polystyrene (s-PS) layer, has been described in an international patent [23] as well as in a recent paper, in which the ability of an active s-PS nanoporous-crystalline layer to prolong the shelf-life of non-climacteric fruits, like oranges, is shown [24]. Moreover, as for s-PS, the ability to prolong the shelf-life of fruit and vegetables, by active films characterized by s-PS co-crystalline phases including antimicrobial guest molecules, has been also described in the literature [21,22].

A previous paper studied the effects of storage temperature, storage duration, and type of film for packaging on some physicochemical properties of red raspberries cv Erika [25]. In this paper, the same variables were applied on the same samples with regards to the phenolic composition and the antioxidant properties.

## 2. Materials and Methods 

### 2.1. Materials

Sampling was described in a previous paper [25]. In brief, the fruits (Red raspberries cv. Erika) were obtained from a greenhouse in Reggio Calabria (Southern Italy) and brought to the laboratory immediately after the harvest, where they were hand packed in different films. 

Two films, nanoactive A (NA) and nanoactive B (NB), with ~50 µm of thickness and one polyethylene terephthalate film with 12 µm of thickness (PET) were used [25]. The nanoactive films are three-layer films (PP/sPS/PP) with overall thickness of nearly 50 µm, made in lab-scale and composed by isotactic polypropylene (PP) and syndiotactic polystyrene (sPS) in the ratio 80:20 (PP/sPS/PP). These films were co-extruded by blown process: after extrusion, the core layer of syndiotactic polystyrene is amorphous (B film), only after a patented treatment [23] the s-PS film core layer is transformed in the disordered nanoporous crystalline phase (A film) [26]. The s-PS used in this study to prepare the nanoactive film was manufactured by Dow Chemical Company (Midland, Michigan, USA), under the trademark Questra 101.

The PET film ≈ 12 µm thick is a Biaxially oriented polyethylene terephthalate film that presents the following characteristics: low permeability to water and O_2_, high resistance to acid and basic compounds, high permeability to alcohols and oils, and high aptitude for food packaging use.

No anti-mould or preservative was used. The packages were sealed using a Multivac Vacuum machine (Tecno Pack, Wolfertschwenden, German). Some packaged samples were placed in the fridge at 1 °C, while others were put in the fridge at the same temperature without film (WF). These were analysed daily until no longer visibly suitable for consumption. Other raspberries were stored in the freezer at −20 °C, using the same packaging materials (NA, NB, PET, and WF), these were analysed monthly for 12 months. A further control sample was left unpackaged at room temperature (RT). 

### 2.2. Methods 

#### 2.2.1. Determination of Ascorbic Acid

The Association of Official Analytical Chemists (AOAC) method was applied [27]. To prepare the extraction solution, 15 g of metaphosphoric acid was weighed and together with 40 mL of acetic acid, was added to 200 mL of deionised water. The mixture was stirred until the acids were dissolved and then made up to volume with deionised water in a 500 mL flask. For the titration, a solution of 50 mg of 2,6-dichloro-indophenol together with 42 mg of sodium bicarbonate in 50 mL of deionised water was prepared. To this, 200 mL of deionised water was added, filtered, and store refrigerated until used. The standard solution of ascorbic acid has a concentration of 1 mg/mL. The sample was obtained by weighing 20 g of fruits, to which 25 mL of deionised water was added, homogenized with Ultra Turrax, centrifuged at 4000 rpm for 10 min, and filtered. Ten mL of centrifuged juice was taken and brought to volume in a 50 mL flask with the extraction solution. At this point, 2 mL of ascorbic acid and 5 mL of the extraction solution were mixed and were titrated until the indicator turned pink for at least 10 s. This is done to determine mg ascorbic acid equivalent to 1 mL of the titrant (a). For the analysis, 5 mL of the sample was added to 5 mL of the extraction solution and was titrated until the colour changed to pink. The vitamin C content expressed in mg/100 g of fruit is given by the following formula:

((a) × V × DF/v) × 100(1)

where: (a): mg of ascorbic acid equivalent to 1 mL of titrant, V: volume of titrant, DF: dilution factor, v: volume of the sample.

#### 2.2.2. Determination of Total Phenolic Content (TPC)

The determination of total polyphenols was conducted as suggested by Slinkard and Singleton [28]. In brief, three grams of fruit was weighed and added to 40 mL of extraction solution consisting of 70% acetone, 29.5% of deionised water, and 0.5% acetic acid. The preparation was left in the dark for 1 h, filtered with filter paper, and put into a Rotavapor at 40 °C to concentrate the sample to 25 mL by evaporating the acetone, and subsequently made up to a volume of 30 mL with deionised water. 

In a 25 mL glass flask: 1 mL of sample, 5 mL of deionised water, and 1 mL of Folin–Ciocalteu reagent was added, and after 1 to 8 min max 10 mL of sodium carbonate was added, after that, the mixture was brought to volume with deionised water and was stored in the dark for 2 h at room temperature. The absorbance was measured in a spectrophotometer at 765 nm. See also the Supplementary Materials.

#### 2.2.3. Determination of Total Monomeric Anthocyanin Content (TMAC)

Preparation of buffer solutions: the method proposed by AOAC was applied [29]. Preparation of the sample: the extraction was carried out by mixing 10 g of fruit in 100 mL solution of water-methanol (1:1), it was homogenized with a blender and filtered under vacuum. All dilutions were carried out in a 50 mL flask. The sample with the maximum concentration was 10 mL (1:4) in order to not exceed the concentration of the buffer. These extracts were used for either the quantification of the total anthocyanins and the individual anthocyanin compounds. 

The measurement of absorbance was done within 20–25 min of preparation.

The measure of the ability of absorption at 700 nm was carried out to correct the errors. However, if the sample is excessively turbid, it must be clarified by centrifugation or filtration prior to the measurement. The concentration of the pigment anthocyanin, expressed in equivalents of cyanidin-3-glucoside, was calculated as follows:

A × MW × DF × 10^3^/e × L (2)

where A = (A_520 nm_ − A_700 nm_) pH 1.0 − (A_520 nm_ − A_700 nm_) pH4.5, MW (molecular weight) = 449.2 g/mol for cyanidin-3-glucoside, DF = dilution factor in the preparation of the sample, L = optical path in cm, e = 26,900 extinction coefficient molar, equal to L × mol^−1^ × cm^−1^, for Cyd-3-glu, and 10^3^ = conversion factor from grams to mg. See also the Supplementary Materials.

#### 2.2.4. Determination of Total Flavonoid Content (TFC) 

The method proposed by Zhishen et al. [30] was applied. One mL of juice and 4 mL of deionised water were mixed, and to this solution, 0.3 mL of NaNO_2_ (sodium nitrate 5%) was added. After 5 min, 0.3 mL of AlCl_3_ (aluminium chloride at 10%) was added, and after 6 min, 2 mL of 1M NaOH was added. It was made up to volume with deionised water in a volumetric flask of 10 mL, and after 15 min, the reading in a spectrophotometer was carried out at 510 nm using a cuvette with an optical path of 1 cm.

#### 2.2.5. Determination of the Antioxidant Activity (AA) by DPPH (2,2-Diphenyl-1-picrylhydrazyl)

The method proposed by Molyneux was applied [31]. A mother solution was prepared from 12.5 mg of ascorbic acid in 25 mL of 13% ethanol-deionised water solution. Then, in a 5 mL flask, four dilutions were prepared: 0.4 mg/mL, 0.3 mg/mL, 0.2 mg/mL, and 0.1 mg/mL. Each solution was made up to volume with the 13% ethanol-deionised water solution. Three mL of DPPH at a concentration of 6 × 10^−5^ M was mixed with 5 µL of sample (or standard) and placed in a cuvette with an optical path of 1 cm, and the reading was carried out in a spectrophotometer from 515 nm at T_0_ to T_5_ min.

#### 2.2.6. Determination of the Individual Anthocyanin Components using the High-Performance Liquid Chromatography (HPLC)

The same extracts used for the quantification of the total anthocyanins, were used for the identification of the individual anthocyanin compounds, but these were filtered with a 0.45 µm filter. The individual anthocyanin compounds were quantified by direct injection of the samples, appropriately diluted in the mobile phase and filtered through a 0.45µm filter (Sartorius Minisart RC-4), in a HPLC system (Knauer Smartline Pump 1000), equipped with a Knauer Smartline UV detector 2600, and using a Knauer column Eurospher 100-5 C18 (150 × 4.6 mm equipped with a guard column). As a mobile phase, acidified water containing 5% formic acid (A) and acetonitrile (B) was used. The flow rate was 1 mL/min, the volume injected was equal to 50 μL, with a gradient profile consisting of A with the following proportions (*v/v*) of B: 0 min, 5%, 1–10 min, 5%–20% B, 10–20 min, 20%–40% B, 20–30 min, 40%–50% B, 30–50 min, 50%–5% B. The system was balanced by maintaining the starting conditions for 10 min between injections. The detector for the acquisition of the UV spectrum was set at 520 nm. The identification of the peaks was carried out by comparing the retention times of the peaks with those of the reference standard. The quantification was carried out by creating calibration curves of the individual pure standards (Extrasyntèse).

Statistical analysis: Analyses were conducted in six replicates. Statistical analysis and Tukey’s test were carried out using SPSS software 22.0 Version (SPSS Inc., Elgin, Illinois, USA). Excel software (Office 2010) (Microsoft Corporation, Redmond, Washington, USA) was used for means and standard deviation calculation, it was also used to generate the charts. 

## 3. Results

For the unpackaged fruits left at room temperature, three days after harvest, strong activity and visible deterioration in quality were observed. Measurements were taken for only two days after harvest.

### 3.1. The Total Phenolic Content (TPC)

In the raspberries stored at room temperature and WF, an initial total phenolic content (TPC) increase (281.22 mg of gallic acid/100 g fw at day 1) and a subsequent decrease (168.31 mg of gallic acid/100 g fw at day 2) was found (Table 1). When packed in the different packaging (film NA, NB, PET, and WF) and stored in the fridge and the freezer, this parameter was influenced by the storage period (*P* ≤ 0.01), (Table 2, Table 3, Table 4, Table 5, Table 6, Table 7, Table 8 and Table 9). TPC decreased during storage both in the fridge and in the freezer. The absolute lowest value was observed in the fruits stored WF after 10 months storage in the freezer (57.96 mg of gallic acid/100 g fw) (Table 9).

In the fruits stored in the fridge, an initial TPC decrease and an increase at days 10–11 for fruits stored with A, PET, and WF was found. In the fruits stored in the freezer (Table 6, Table 7, Table 8 and Table 9), the worst performance was found in red raspberries WF, where in three cases, less than 80 mg of gallic acid/100 g fw was found, and in 5 cases, less than 100 mg of gallic acid/100 g fw was found. TPC showed high significant differences (*P* < 0.01) if the considered variables are film x days (refrigerated fruits) and film x months (frozen fruits). No significant differences were found in all other cases (Table 10). 

### 3.2. Total Monomeric Anthocyanin Content (TMAC)

The total monomeric anthocyanin content (TMAC) was low at harvest (19.10 mg of cyanidin-3-O-glucoside/100 g fw) and rapidly increased up to 83.55 mg of cyanidin-3-O-glucoside/100 g fw, i.e., 337% more when at RT and WF (Table 1). The same increasing trend was observed in the fruits stored in the fridge under the studied conditions (*P* < 0.01) (Table 2, Table 3, Table 4 and Table 5) but the highest values were observed in the fruits stored WF in the fridge. In this condition, the TMAC varied from 25.08 to 94.37 mg of cyanidin-3-O-glucoside/100 g fw from day 1 to day 10 (Table 5).

The lowest values during storage in the fridge were found in raspberries wrapped with the film NB, varying from 18.15 (day 1) to 42.73 mg of cyanidin-3-O-glucoside/100 g fw (day 7) (Table 3). The TMAC increased 437% in two days (at RT and WF), 318% in 14 days (in the fridge, film NA), 235% in 7 days (in the fridge, film NB), 261% in 12 days (in the fridge, film PET), and 376% (in the fridge and WF). The raspberries stored in the freezer showed a significantly different TMAC and an increasing trend (*P* < 0.01) with film NA, film NB, and film PET (Table 6, Table 7 and Table 8), whereas no significant differences were found when fruits were stored WF (Table 9). 

Table 10 shows that the TMAC was not influenced by the film effect and by the interaction (film × months) when fruits were stored in the freezer, but it was highly influenced (*P <* 0.01) by film, storage period (days), interaction between film and days in the fridge, and by the storage period (months) in the freezer.

### 3.3. Total Flavonoid Content (TFC)

The total flavonoid content (TFC) of red raspberries was influenced by the storage period, using the different packaging methods, *P* < 0.01 for film NA, in the fridge and freezer: *P* < 0.05 for film NB in the fridge and *P* < 0.01 for film NB in the freezer, *P* < 0.01 for film PET, in the fridge and freezer, *P* < 0.01 for storage WF, in the fridge and freezer. The fruits stored at room temperature and WF showed a trend in TFC similar to TPC with an initial increase and a fall at the second and last day of storage from 27.06 mg of catechin/100g fw (at harvest) to 47.14 mg of catechin/100g fw (one day storage), to 20.31 mg of catechin/100g fw to 47.14 (two days storage) (Table 1).

TFC of fruits wrapped with film NA showed a tendency to decrease during 14 days of storage in the fridge with significant differences day by day (*P* < 0.01) (Table 2). The storage duration in the fridge also affected the TFC in raspberries wrapped with film NB (*P* < 0.05), film PET (*P* < 0.01), and WF (*P* < 0.01) (Table 2, Table 3, Table 4 and Table 5).

When the fruits were stored in the freezer, the TFC showed an initial increase and a decline after the third month of storage and a high significant different content during storage (*P* < 0.01) (Table 6, Table 7, Table 8 and Table 9). 

The TFC was influenced neither by the packaging methods, nor by the interaction between film and days or film and months, but it was highly influenced (*P <* 0.01) by the storage period (fridge or freezer), (Table 10).

### 3.4. Ascorbic Acid Content

Ascorbic acid decreased rapidly in two days of storage at RT and WF from 42.25 to 28.49 mg of ascorbic acid/100g of fw, i.e., 32.56% less (Table 1).

The highest value observed for this parameter (42.25 mg of ascorbic acid/100 g fw) was observed at harvest (Table 1). Ascorbic acid content decreased during storage and the lowest value was observed in the fruits stored in the freezer, in the film NB, 22.42 mg of ascorbic acid/100 g fw. For the storage in the fridge, this content decreased after the third day of storage for NA, PET, and WF. For NB, it was after the fourth day. For the storage in the freezer, the content decreased after the fourth month of storage. The ascorbic acid content was not influenced by the storage period in the fridge without film but was highly influenced by the storage period (fridge or freezer), using the different packaging methods (*P <* 0.01). 

In Table 10, it is shown that this parameter is not influenced by the packaging method in either ways of storage (fridge or freezer) and also it was not influenced by the storage period in the fridge, but it was influenced by the interaction film, the storage period in the fridge (days), and the interaction month x films (*P <* 0.01).

### 3.5. The Antioxidant Activity (AA), (DPPH assay)

The antioxidant activity (AA) measured in red raspberries stored at RT and WF increased rapidly during storage, in particular the DPPH assay value was more than double in two days from 102.46 to 207.00 mg ascorbic acid equivalent (AAE)/100g fw. Apparently, in RT stored fruits, this trend could be related to the TMAC (Table 1). A partially different behaviour was found when fruits were stored in the fridge and in the freezer, this could be due to the influence of refrigeration and freezing which modify the physiological evolution of raspberries if compared with the ones stored at RT (Table 2, Table 3, Table 4, Table 5, Table 6, Table 7, Table 8 and Table 9). The AA values showed high significant differences (*P* < 0.01) in all the studied conditions (Table 2, Table 3, Table 4, Table 5, Table 6, Table 7, Table 8 and Table 9). The AA trend in the fruits stored in the fridge was partially constant, this was related to the different trends of the different classes of antioxidants found in the raspberries and by the different influence of each class of antioxidant (Table 2, Table 3, Table 4 and Table 5). In addition, it was also related to each single antioxidant contained (Table 6, Table 7, Table 8 and Table 9) in the raspberry fruit in the DPPH assay value. In fruits stored in the fridge and wrapped with film NA (Table 2), the two highest AA values were found (194.61 and 176.61 mg AAE/100g fw) respectively on the fourth and the fifth days of storage. In particular, the highest AA value on the fourth day was in agreement with a high TMAC (48.50 mg cyanidin-3-O-glucoside/100 g fw) and with the highest ascorbic acid content (34.93 mg/100 g fw). In the fruits stored with film NB, the two highest AA values were found in the fruits on the third (206.57 mg AAE/100g fw) and the sixth day (204.63 mg AAE/100g fw) of storage in the fridge (Table 3). On fruits stored in the fridge with film PET, lower values were generally found (Table 4 and Table 5) than in fruits wrapped with film NA and NB. The low temperature used in the freezer storage negatively influenced the AA (Table 6, Table 7, Table 8 and Table 9), in fact, all values were lower than 87.81 mg AAE/100g fw found in fruits wrapped with film PET in the fourth month of storage (Table 8). The fourth month also presented the highest AA value in the frozen fruits stored WF (Table 9). If the AA mean values of the different packaging are compared between fridge and freezer conditions, one can see that AA value of fruits wrapped with film NA in the fridge condition are 253% higher than the ones wrapped with the same film but stored in the freezer (Table 10). If film NB is considered, the AA observed for the mean values of fruits stored in the fridge are 392% higher than those of the fruits stored in the freezer. In the same way, the AA value of fruits stored in the fridge with film PET were 258% higher than in the fruits stored in the freezer and AA value of fruits stored in the fridge WF prevailed (284% more) on AA value of fruits stored in the freezer WF (Table 10). By comparing the different packaging methods, AA was found to be influenced by the storage period for film NA (*P <* 0.01 in the fridge and *P <* 0.05 in the freezer), *P <* 0.01 for film NB (fridge and freezer), *P <* 0.01 for film PET (fridge and freezer), and *P <* 0.01 for WF (fridge and freezer) (Table 2, Table 3, Table 4, Table 5, Table 6, Table 7, Table 8 and Table 9). 

The two-way ANOVA applied on the AA showed that film variable was not significant in either the refrigerated or frozen fruits and neither in the interaction films x months, whereas the period of storage had a significant influence (*P* < 0.05) in the refrigerated fruits and a high significant influence (*P* < 0.01) in the frozen ones (Table 10). 

### 3.6. The Individual Anthocyanidin Compounds Observed in the UV-HPLC Instrument

In the red raspberries of the present study, four individual anthocyanidin compounds were identified using the HPLC instrument: cyanidin-3-O-sophoroside, cyanidin-3-O-glucoside, cyanidin-3-O-rutinoside, and pelargonidin-3-O-glucoside, as it is shown in Table 11, in which the content of the individual anthocyanidin compounds at harvest is described. All these compounds were highly influenced *P <* 0.01 by the packaging method when stored in the fridge but were not influenced by this effect when stored in the freezer (Table 10). In the same table it is shown that the cyanidin-3-O-sophoroside, cyanidin-3-O-glucoside were highly influenced *P <* 0.01 by the storage period in the fridge and the freezer, unlike the two other compounds cyanidin-3-O-rutinoside and pelargonidin-3-O-glucoside, which were not influenced by the storage period in the fridge but highly influenced by the storage duration in the freezer *P <* 0.01. 

When the red raspberries were stored in the fridge, there were significant differences between days for almost all the parameters, except for cyanidin-3-O-glucoside and cyanidin-3-O-rutinoside (Table 10). 

When the red raspberries were packed and stored at −20 °C in the freezer, they showed good conditions and there was no significant difference between months for the cyanidin-3-O-rutinoside packed with NB (Table 12) and WF (Table 13).

Cyanidin-3-O-sophoroside was not influenced by the storage period in the fridge, when packed in the film NB (Table 14) and WF (Table 15), but it was influenced when packed in the other packaging methods, in the fridge or the freezer (Table 11, Table 12 and Table 13, Table 16, Table 17 and Table 18). The highest value for this parameter was observed in the fruits stored WF in the fridge 81.42 µg/g of fw (Table 15) and the lowest was observed in fruits stored WF in the freezer 41.61 µg/g of fw (Table 13).

The cyanidin-3-O-glucoside was not influenced by the storage period in the fridge when packed in the films NA, NB, and WF (Table 14, Table 19, Table 15). Compared to the value at harvest 55.84 µg/g of fw (Table 10), the lowest value was observed in the film NB stored for 10 months in the freezer 33.07 µg/g of fw (Table 17).

When the fruits were stored in the fridge and packed in NB film, all the individual anthocyanidin compounds were not influenced by the days (Table 19), but when fruits were stored in the freezer with NB film, all the individual anthocyanidin compounds were influenced (*P* < 0.01), (Table 17).

The cyanidin-3-O-rutinoside was influenced by the storage period in the fridge (*P* < 0.01) when packed in the film PET (Table 16), and when stored in the freezer when packed in film NB and PET (*P* < 0.01) (Table 16 and Table 17). With regards to this parameter, the lowest value was observed in the frozen fruits (10 months) WF 42.24 µg/g of fw (Table 17).

The pelargonidin-3-O-glucoside was influenced by the storage period (*P* < 0.01) in the fridge only when packed in the film NA (Table 14) and it was influenced by packaging when fruits were stored in the freezer and wrapped with film NA (*P* < 0.05) (Table 12), and with film NB, film PET, and WF (*P* < 0.01) (Table 13, Table 17, Table 18), but it was not influenced (*P* > 0.01) when raspberries were stored in the fridge and wrapped by B, PET, and WF (Table 15, Table 16, Table 19).

## 4. Discussion

When the experiment was conducted on fruits stored in the fridge, the longest shelf-life (14 days) was found with raspberries packed in the film NA, the material which maintains the antioxidant characteristics of these fruits, in good condition, for the longest period of time. When the fruits were frozen, the packaging method did not influence all the parameters, however almost all parameters were highly influenced by the storage period with the exception of the total phenolic content (Table 10). When raspberries were stored in the fridge, the storage period did not influence the ascorbic acid content, cyanidin-3-O-rutinoside, and pelargonidin-3-O-glucoside.

Unlike the NA film, the shelf-life of the red raspberries when packed in the film NB and stored in the fridge was maintained for seven days (one half compared to NA). On the seventh day, the appearance of the fruits was not appealing and from the commercial point of view, these fruits could not be sold. Even though these fruits lost much water and thus their firmness, no fungi activity was observed using this film either. 

Ascorbic acid (vitamin C) is a water soluble and antioxidant molecule whose ingestion is necessary by humans, due to their impossibility to synthesize it [32]. Dietary sources of vitamin C are fruits and vegetables: especially citrus fruits, cauliflower, and broccoli (i.e., cruciferous plants), tomato juice, cranberry [33], and kiwifruit. Ninety mg/day for adult men and 75 mg/day for adult women are the doses recommended by the National Institute of Health [34]. In the red raspberries of our study and stored at RT and in the fridge, a higher ascorbic acid content with respect to frozen fruits was found (Table 2, Table 3, Table 4, Table 5, Table 6, Table 7, Table 8 and Table 9). The lowest vitamin C content (10.83 mg of ascorbic acid/100 g of fresh weight) was found on the tenth day of storage in the freezer and WF (Table 9), this means that 100g of frozen fruits can give 1/7 to 1/9 of the daily recommended vitamin C. 

Pre- and post-harvest environmental conditions, such as temperature and light, influence the anthocyanin and total phenolic concentration in fruits [35]. Phenolics are antioxidant compounds which have the ability to reduce pro-oxidant agents. Similar to our findings, Çekiçand Özgen [36] studied 22 wild accessions and 2 cultivars (Heritage and Tulameen) in Turkey and found a TPC content varying between 149 and 348 mg/100 g fw, and both the highest and the lowest value were revealed in the wild accessions. Flavonoids are phenolic compounds that accumulate in plant tissues and include flavonols, flavones, flavan-3-ols, flavanones and chalcones, anthocyanins, and isoflavones. Their colour varies from white and yellow (flavonols) to red and blue (anthocyanins).

Anthocyanins are responsible for the blue, purple, red, and orange colours of fruits and vegetables [37]. They were proved to prevent or lower the risk of cancer [38], diabetes [39], and cardiovascular diseases [40]. The degree to which anthocyanins exert their bioactive functions is in relation to their chemical structure, in particular by types, number, and position of substitutions [41,42,43]. In the literature, more than 600 different anthocyanins isolated from plants are described [44], whose de-glycosylated or aglycone forms are classified as anthocyanidins [37]. Seventeen anthocyanidins were found in nature, and 6 out of 17 (i.e., cyanidin, delphinidin, petunidin, peonidin, pelargonidin, and malvidin, with cyanidin being the most common) are widely found [45]. As per our findings, the total anthocyanin contents increased significantly with storage. Anthocyanic content is regulated by the intensity of light exposure [46], for this reason, the highest TMAC (83.55 mg of gallic acid/100 g of fresh weight) was reached in two days in red raspberries stored at RT (Table 2). Also, low temperature induced anthocyanins accumulation [47] and in our study we found an increasing anthocyanic accumulation trend, more evident in raspberries stored in the fridge than in frozen ones (Table 3, Table 4, Table 5, Table 6, Table 7, Table 8 and Table 9). Increases in anthocyanin content during storage have also been reported for strawberries [48], lowbush blueberries [49,50], rabbiteye blueberries [51], and raspberries [52].

The synthesis of both anthocyanins and non-anthocyanins may have contributed to the increase in antioxidant activity in red raspberries after storage. Following the pink stage, many phytonutrients are synthesized in parallel with the overall development and maturation of the fruit. The fully mature red raspberries have stronger antioxidant activities [11]. 

During storage, decreases in titratable acidity may provide carbon skeletons for the synthesis of phenolics, including both anthocyanin and non-anthocyanin phenolics [52]. 

DPPH assay is used to determine the antioxidant potential of many food matrices and plant extracts such as edible vegetable oils [53,54,55] and juice fruits [24,56,57]. DPPH assay values are generally related with the total flavonoid content [58,59], and a positive correlation trend can be observed in the refrigerated and frozen red raspberries studied here, in fact, in DPPH, assay values decrease when total flavonoid content decreases (Table 3, Table 4, Table 5, Table 6, Table 7, Table 8 and Table 9), whereas in the fruit stored at RT, this trend was partially influenced by the high TMAC on the second day of storage (Table 2). 

## 5. Conclusions

The bioactive compounds that were detected in these fruits, using the different packaging materials and storage methods, were at very high levels. For raspberries stored in the fridge, the use of nanoactive A and polyethylene terephthalate prolonged and preserved the antioxidant properties of fruits compared to fruits stored without film or wrapped with nanoactive B. The fruits stored in the nanoactive film A showed better performance regarding storage in the fridge or freezer. In the fridge (1 °C), the shelf life of the red raspberries, when packed in the nanoactive A film, lasted longer (14 days). The best performance of Nanoactive A is probably due to the high barrier action offered by this film to O_2_ diffusivity as this gas accelerates the senescence of climacteric fruits. One-year storage of raspberries in the freezer maintained the antioxidant properties of red raspberries.

## Figures and Tables

**Table 1:** Fruits left at room temperature without film. Effect of storage period, from harvest on the total phenolic content, total monomeric anthocyanin content, total flavonoid content, the ascorbic acid content, and the antioxidant activity DPPH parameters in red raspberries (cv. Erika). The results are the mean of three replicates ± Standard Deviation. The letters represent the significant difference vertically; ** (*P* ≤ 0.01). The letters represent the significant difference vertically at *P* ≤ 0.05.

Day	Total Phenolic Content ^1^	Total Monomeric Anthocyanin Content ^2^	Total Flavonoids Content ^3^	Ascorbic Acid Content ^4^	AA DPPH Assay ^5^
**0**	187.63 ± 32.20 ^a^	19.10 ± 0.43 ^a^	27.06 ± 3.68 ^ab^	42.25 ± 4.57 ^b^	102.46 ± 7.61 ^a^
**1**	281.22 ± 48.71 ^b^	23.34 ± 1.82 ^a^	47.14 ± 15.74 ^b^	33.34 ± 4.16 ^ab^	111.22 ± 19.23 ^a^
**2**	168.31 ± 6.81 ^a^	83.55 ± 7.21 ^b^	20.31 ± 1.22 ^a^	28.49 ± 1.52 ^a^	207.00 ± 46.59 ^b^
**Significance**	**	**	**	**	**

^1^ Results are expressed as mg of gallic acid/100 g of fresh weight; ^2^ Results are expressed as mg of cyanidin-3-O-glucoside/100 g of fresh weight; ^3^ Results are expressed as mg of catechin/100 g of fresh weight; ^4^ Results are expressed as mg of ascorbic acid/100 g of fresh weight; ^5^ Results are expressed as mg of ascorbic acid/100 g of fresh weight.

**Table 2:** Fruits stored at 1 °C in fridge, packaged in Nanoactive A film. Effect of storage period, on the total phenolic content, total monomeric anthocyanin content, total flavonoid content, the ascorbic acid content, and the antioxidant activity DPPH parameters in red raspberries (cv. Erika). The results are the mean of three replicates ± Standard Deviation. ** (*P* ≤ 0.01); *** (*P* ≤ 0.001). The letters represent the significant difference vertically at *P* ≤ 0.05.

Day	Total Phenolic Content ^1^	Total Monomeric Anthocyanin Content ^2^	Total Flavonoids Content ^3^	Ascorbic Acid Content ^4^	AA DPPH Assay ^5^
**1**	217.15 ± 24.12 ^e^	15.96 ± 2.45 ^ab^	33.92 ± 7.85 ^a^	28.72 ± 1.61 ^bcde^	151.34 ± 24.29 ^bc^
**2**	131.94 ± 20.99 ^ab^	36.43 ± 11.35 ^a^	16.21 ± 2.66 ^bc^	27.93 ± 0.76 ^bcd^	154.90 ± 29.68 ^bc^
**3**	138.71 ± 16.13 ^abc^	36.74 ± 4.84 ^a^	13.32 ± 2.79 ^bc^	23.19 ± 0.12 ^ab^	164.84 ± 21.86 ^bc^
**4**	124.07 ± 12.31 ^a^	48.50 ± 6.28 ^a^	17.25 ± 1.36 ^de^	34.93 ± 2.75 ^ef^	194.61 ± 10.06 ^c^
**5**	166.72 ± 13.15 ^bcd^	47.14 ± 1.36 ^ab^	22.64 ± 2.56 ^cd^	30.47 ± 2.17 ^cde^	176.61 ± 7.27 ^c^
**6**	130.49 ± 14.83 ^ab^	46.82 ± 1.72 ^bc^	21.70 ± 2.54 ^b^	26.87 ± 1.52 ^bcd^	107.03 ± 13.58 ^b^
**7**	147.74 ± 18.04 ^abcd^	43.72 ± 5.37 ^a^	18.11 ± 3.58 ^bcd^	32.41 ± 4.72 ^cdef^	86.92 ± 26.78 ^a^
**8**	151.79 ± 17.56 ^abcd^	58.90 ± 2.69 ^a^	17.02 ± 1.77 ^e^	26.42 ± 4.81 ^bc^	77.93 ± 13.08 ^a^
**9**	150.81 ± 9.97 ^abcd^	43.77 ± 2.04 ^a^	13.60 ± 1.79 ^bcd^	19.88 ± 0.76 ^a^	86.07 ± 7.66 ^a^
**10**	185.57 ± 21.26 ^de^	41.09 ± 1.95 ^a^	18.45 ± 3.30 ^bcd^	23.10 ± 1.52 ^ab^	91.53 ± 10.53 ^a^
**11**	172.69 ± 16.98 ^cd^	23.05 ± 4.86 ^ab^	21.83 ± 5.21 ^a^	30.01 ± 4.17 ^cde^	71.42 ± 14.38 ^a^
**12**	174.05 ± 20.88 ^cd^	34.60 ± 7.42 ^ab^	21.79 ± 4.15 ^b^	36.90 ± 2.54 ^f^	64.93 ± 11.31 ^a^
**13**	153.57 ± 13.90 ^abcd^	43.05 ± 2.24 ^a^	18.60 ± 0.29 ^bcd^	33.26 ± 2.02 ^def^	87.90 ± 14.92 ^a^
**14**	166.70 ± 5.87 ^bcd^	50.82 ± 3.07 ^c^	24.64 ± 3.67 ^de^	32.42 ± 3.26 ^cdef^	95.93 ± 3.24 ^a^
**Significance**	**	**	**	**	***

^1^ Results are expressed as mg of gallic acid/100 g of fresh weight; ^2^ Results are expressed as mg of cyanidin-3-O-glucoside/100 g of fresh weight; ^3^ Results are expressed as mg of catechin/100 g of fresh weight; ^4^ Results are expressed as mg of ascorbic acid/100 g of fresh weight; ^5^ Results are expressed as mg of ascorbic acid/100 g of fresh weight.

**Table 3:** Fruits stored at 1 °C in fridge, packaged in Nanoactive B film. Effect of storage period, on the total phenolic content, total monomeric anthocyanin content, total flavonoid content, the ascorbic acid content, and the antioxidant activity DPPH parameters in red raspberries (cv. Erika). The results are the mean of three replicates ± Standard Deviation. *** (*P* ≤ 0.001); ** (*P* ≤ 0.01); * (*P* ≤ 0.05). The letters represent the significant difference vertically at *P* ≤ 0.05.

Day	Total Phenolic Content ^1^	Total Monomeric Anthocyanin Content ^2^	Total Flavonoids Content ^3^	Ascorbic Acid Content ^4^	AA DPPH Assay ^5^
**1**	235.26 ± 42.12 ^b^	18.15 ± 1.33 ^a^	23.84 ± 0.44 ^c^	21.52 ± 0.80 ^a^	131.11 ± 8.27 ^b^
**2**	170.93 ± 3.88 ^a^	26.73 ± 1.76 ^bc^	21.74 ± 0.63 ^abc^	25.27 ± 0.77 ^ab^	126.91 ± 16.63 ^b^
**3**	164.39 ± 13.12 ^a^	42.24 ± 1.59 ^d^	19.89 ± 0.58 ^ab^	21.80 ± 0.84 ^a^	206.57 ± 10.72 ^c^
**4**	134.79 ± 7.51 ^a^	22.73 ± 2.38 ^b^	16.84 ± 1.60 ^a^	35.47 ± 5.27 ^d^	188.43 ± 10.10 ^c^
**5**	160.28 ± 30.13 ^a^	31.45 ± 1.57 ^c^	18.42 ± 2.92 ^ab^	33.67 ± 2.22 ^cd^	73.39 ± 14.67 ^a^
**6**	141.97 ± 2.95 ^a^	39.05 ± 2.13 ^d^	23.45 ± 1.11 ^bc^	26.87 ± 2.75 ^abc^	74.63 ± 8.67 ^a^
**7**	171.87 ± 18.46 ^a^	42.73 ± 2.53 ^d^	19.37 ± 2.66 ^ab^	30.70 ± 5.11 ^bcd^	58.05 ± 10.35 ^a^
**Significance**	**	**	*	**	***

^1^ Results are expressed as mg of gallic acid/100 g of fresh weight; ^2^ Results are expressed as mg of cyanidin-3- O-glucoside/100 g of fresh weight; ^3^ Results are expressed as mg of catechin/100 g of fresh weight; ^4^ Results are expressed as mg of ascorbic acid/100 g of fresh weight; ^5^ Results are expressed as mg of ascorbic acid/100 g of fresh weight.

**Table 4:** Fruits stored at 1 °C in fridge, packaged in PET (polyethylene terephthalate) film. Effect of storage period on the total phenolic content, total monomeric anthocyanin content, total flavonoid content, the ascorbic acid content, and the antioxidant activity DPPH parameters in red raspberries (cv. Erika). The results are the mean of three replicates ± Standard Deviation. ** (*P* ≤ 0.01). The letters represent the significant difference vertically at *P* ≤ 0.05.

Day	Total Phenolic Content ^1^	Total Monomeric Anthocyanin Content ^2^	Total Flavonoids Content ^3^	Ascorbic Acid Content ^4^	AA DPPH Assay ^5^
**1**	236.49 ± 5.41 ^d^	16.88 ± 1.91 ^e^	32.46 ± 2.51 ^a^	42.89 ± 1.26 ^f^	104.48 ± 4.30 ^b^
**2**	136.39 ± 5.82 ^a^	57.94 ± 8.72 ^a^	13.89 ± 0.72 ^bc^	26.87 ± 0.77 ^bcd^	147.97 ± 3.93 ^c^
**3**	161.17 ± 6.45 ^ab^	43.83 ± 6.49 ^cd^	22.59 ± 2.06 ^b^	23.56 ± 0.79 ^ab^	175.62 ± 18.31 ^d^
**4**	134.02 ± 12.38 ^a^	64.07 ± 4.37 ^ab^	14.36 ± 1.31 ^c^	34.39 ± 2.02 ^e^	76.68 ± 9.62 ^a^
**5**	158.31 ± 24.39 ^ab^	59.79 ± 8.17 ^bcd^	20.06 ± 4.69 ^bc^	31.53 ± 1.51 ^de^	84.00 ± 3.88 ^ab^
**6**	162.11 ± 26.14 ^ab^	68.47 ± 11.13 ^bcd^	20.29 ± 4.05 ^c^	25.25 ± 0.77 ^bc^	86.68 ± 3.69 ^ab^
**7**	158.89 ± 7.96 ^ab^	53.85 ± 10.52 ^bcd^	20.21 ± 0.45 ^bc^	30.33 ± 1.54 ^cde^	82.93 ± 9.73 ^ab^
**8**	177.45 ± 17.52 ^bc^	66.31 ± 2.23 ^cd^	23.84 ± 1.52 ^c^	31.25 ± 4.20 ^cde^	89.38 ± 7.01 ^ab^
**9**	151.96 ± 14.46 ^ab^	61.02 ± 8.54 ^abc^	18.17 ± 3.72 ^bc^	19.35 ± 2.29 ^a^	81.48 ± 2.96 ^a^
**10**	175.49 ± 14.30 ^bc^	55.07 ± 10.75 ^cd^	22.31 ± 1.92 ^bc^	30.10 ± 2.75 ^cde^	91.81 ± 4.76 ^ab^
**11**	182.42 ± 9.76 ^bc^	60.36 ± 6.09 ^de^	25.17 ± 1.25 ^bc^	32.57 ± 3.15 ^de^	87.15 ± 9.83 ^ab^
**12**	206.30 ± 12.20 ^cd^	44.07 ± 5.25 ^de^	26.15 ± 0.98 ^b^	29.97 ± 1.55 ^cde^	101.59 ± 4.02 ^b^
**Sig.**	**	**	**	**	**

^1^ Results are expressed as mg of gallic acid/100 g of fresh weight; ^2^ Results are expressed as mg of cyanidin-3-O- glucoside/100 g of fresh weight; ^3^ Results are expressed as mg of catechin/100 g of fresh weight; ^4^ Results are expressed as mg of ascorbic acid/100 g of fresh weight; ^5^ Results are expressed as mg of ascorbic acid/100 g of fresh weight.

**Table 5:** Fruits stored at 1 °C in fridge, without packaging film. Effect of storage period on the total phenolic content, total monomeric anthocyanin content, total flavonoid content, the ascorbic acid content, and the antioxidant activity DPPH parameters in red raspberries (cv. Erika). The results are the mean of three replicates ± Standard Deviation. ** (*P* ≤ 0.01); n.s. (not significant). The letters represent the significant difference vertically at *P* ≤ 0.05.

Day	Total Phenolic Content ^1^	Total Monomeric Anthocyanin Content ^2^	Total Flavonoids Content ^3^	Ascorbic Acid Content ^4^	AA DPPH Assay ^5^
**1**	240.37 ± 4.71 ^cd^	25.08 ± 0.30 ^e^	31.29 ± 1.72 ^a^	26.25 ± 2.57 ^a^	86.73 ± 2.82 ^a^
**2**	159.01 ± 3.14 ^ab^	66.40 ± 6.99 ^ab^	16.26 ± 0.91 ^b^	27.41 ± 2.63 ^a^	167.90 ± 10.20 ^b^
**3**	148.91 ± 7.58 ^a^	62.39 ± 14.94 ^ab^	16.20 ± 1.25 ^b^	26.84 ± 2.95 ^a^	87.48 ± 11.10 ^a^
**4**	188.51 ± 17.73 ^ab^	71.99 ± 7.33 ^bc^	20.93 ± 1.86 ^b^	25.26 ± 2.01 ^a^	85.10 ± 11.74 ^a^
**5**	185.14 ± 15.40 ^ab^	74.52 ± 2.68 ^c^	22.07 ± 2.26 ^bc^	29.98 ± 2.00 ^a^	79.88 ± 8.00 ^a^
**6**	150.89 ± 2.13 ^a^	73.25 ± 8.02 ^a^	13.00 ± 2.38 ^bc^	28.48 ± 3.31 ^a^	85.89 ± 10.56 ^a^
**7**	164.52 ± 10.08 ^ab^	80.95 ± 9.52 ^c^	22.11 ± 2.23 ^bc^	27.29 ± 3.26 ^a^	87.94 ± 8.25 ^a^
**8**	172.81 ± 11.72 ^ab^	80.16 ± 8.18 ^bc^	21.00 ± 1.74 ^bc^	26.32 ± 2.78 ^a^	96.16 ± 5.67 ^a^
**9**	183.25 ± 9.32 ^ab^	78.50 ± 6.47 ^c^	22.39 ± 1.74 ^bc^	26.87 ± 1.52 ^a^	102.86 ± 6.24 ^a^
**10**	216.93 ± 14.38 ^b^	94.37 ± 14.27 ^d^	28.35 ± 3.44 ^c^	27.41 ± 2.28 ^a^	87.96 ± 8.27 ^a^
**Sig.**	**	**	**	n.s.	**

^1^ Results are expressed as mg of gallic acid/100 g of fresh weight; ^2^ Results are expressed as mg of cyanidin-3-glucoside/100 g of fresh weight; ^3^ Results are expressed as mg of catechin/100 g of fresh weight; ^4^ Results are expressed as mg of ascorbic acid/100 g of fresh weight; ^5^ Results are expressed as mg of ascorbic acid/100 g of fresh weight.

**Table 6:** Fruits stored at –20 °C in freezer, packaged in Nanoactive A film. Effect of storage period on the total phenolic content, total monomeric anthocyanin content, total flavonoid content, the ascorbic acid content, and the antioxidant activity DPPH parameters in red raspberries (cv. Erika). The results are the mean of three replicates ± Standard Deviation. ** (*P* ≤ 0.01); * (*P* ≤ 0.05). The letters represent the significant difference vertically at *P* ≤ 0.05.

Month	Total Phenolic Content ^1^	Total Monomeric Anthocyanin Content ^2^	Total Flavonoids Content ^3^	Ascorbic Acid Content ^4^	AA DPPH Assay ^5^
**1**	166.65 ± 4.15 ^bc^	39.48 ± 10.64 ^bc^	16.25 ± 3.10 ^d^	30.44 ± 0.59 ^d^	60.03 ± 3.33 ^d^
**2**	168.65 ± 4.15 ^bc^	45.32 ± 8.16 ^c^	19.25 ± 3.10 ^e^	44.12 ± 6.79 ^e^	70.35 ± 18.5 ^e^
**3**	179.70 ± 2.88 ^c^	48.94 ± 6.12 ^c^	23.27 ± 0.51 ^f^	25.12 ± 4.24 ^cd^	52.49 ± 7.19 ^bc^
**4**	153.69 ± 36.86 ^abc^	33.20 ± 3.42 ^bc^	19.91 ± 9.08 ^e^	32.41 ± 3.48 ^d^	55.10 ± 9.81 ^bc^
**5**	196.20 ± 55.52 ^c^	35.41 ± 11.94 ^bc^	11.76 ± 0.74 ^b^	32.77 ± 1.09 ^d^	41.60 ± 24.9 ^ab^
**6**	115.13 ± 1.62 ^a^	43.33 ± 9.63 ^c^	8.72 ± 0.77 ^a^	17.91 ± 2.50 ^ab^	20.25 ± 6.81 ^a^
**7**	156.69 ± 38.96 ^abc^	25.62 ± 4.52 ^ab^	7.87 ± 2.68 ^a^	22.23 ± 2.86 ^bc^	15.05 ± 1.69 ^a^
**8**	106.06 ± 11.16 ^ab^	17.29 ± 2.00 ^a^	14.92 ± 2.78 ^c^	12.97 ± 1.33 ^a^	43.97 ± 5.36 ^ab^
**9**	97.74 ± 38.37 ^a^	33.51 ± 1.71 ^bc^	8.32 ± 5.02 ^a^	14.86 ± 0.72 ^ab^	30.39 ± 10.9 ^a^
**10**	107.78 ± 10.49 ^ab^	39.40 ± 1.89 ^bc^	13.37 ± 19.02 ^c^	15.27 ± 0.73 ^ab^	21.66 ± 6.21 ^a^
**11**	145.08 ± 21.41 ^abc^	48.41 ± 2.18 ^c^	20.86 ± 3.84 ^e^	25. 99 ± 5.56 ^cd^	20.62 ± 7.24 ^a^
**12**	146.17 ± 20.33 ^abc^	49.12 ± 2.11 ^c^	19.97 ± 3.22 ^e^	26. 02 ± 4.44 ^cd^	19.90 ± 6.16 ^a^
**Sig.**	**	**	**	**	*

^1^ Results are expressed as mg of gallic acid/100 g of fresh weight; ^2^ Results are expressed as mg of cyanidin-3-O- glucoside/100 g of fresh weight; ^3^ Results are expressed as mg of catechin/100 g of fresh weight; ^4^ Results are expressed as mg of ascorbic acid/100 g of fresh weight; ^5^ Results are expressed as mg of ascorbic acid/100 g of fresh weight.

**Table 7:** Fruits stored at –20 °C in freezer, packaged in Nanoactive B film. Effect of storage period on the total phenolic content, total monomeric anthocyanin content, total flavonoid content, the ascorbic acid content, and the antioxidant activity DPPH parameters in red raspberries (cv. Erika). The results are the mean of three replicates ± Standard Deviation. ** (*P* ≤ 0.01). The letters represent the significant difference vertically at *P* ≤ 0.05.

Month	Total Phenolic Content ^1^	Total Monomeric Anthocyanin Content ^2^	Total Flavonoids Content ^3^	Ascorbic Acid Content ^4^	AA DPPH Assay ^5^
**1**	164.72 ± 7.57 ^cde^	25.26 ± 1.63 ^a^	14.77 ± 3.24 ^b^	28.63 ± 2.34 ^def^	70.30 ± 5.84 ^e^
**2**	166.72 ± 7.57 ^de^	24.46 ± 1.28 ^a^	17.77 ± 3.24 ^b^	34.84 ± 3.00 ^ef^	47.15 ± 12.07 ^c^
**3**	180.16 ± 19.30 ^e^	39.20 ± 2.87 ^bc^	27.05 ± 3.25 ^c^	27.05 ± 3.57 ^cde^	59.32 ± 1.47 ^d^
**4**	137.65 ± 23.87 ^bcd^	35.84 ± 8.43 ^b^	15.28 ± 2.67 ^b^	35.96 ± 10.05 ^f^	45.95 ± 1.56 ^c^
**5**	186.35 ± 7.72 ^e^	23.34 ± 10.30 ^a^	7.53 ± 2.16 ^a^	25.18 ± 0.88 ^bcd^	26.93 ± 5.54 ^b^
**6**	105.11 ± 1.29 ^a^	42.01 ± 4.98 ^cd^	6.63 ± 1.02 ^a^	19.40 ± 3.57 ^abc^	24.47 ± 0.99 ^ab^
**7**	138.71 ± 11.72 ^bcd^	24.67 ± 1.86 ^a^	7.49 ± 0.43 ^a^	18.40 ± 2.52 ^ab^	23.26 ± 2.00 ^ab^
**8**	115.56 ± 11.20 ^ab^	49.82 ± 1.88 ^d^	16.88 ± 1.31 ^b^	12.74 ± 2.24 ^a^	42.56 ± 2.42 ^c^
**9**	109.75 ± 17.06 ^ab^	32.54 ± 0.83 ^ab^	8.58 ± 2.29 ^a^	15.63 ± 1.08 ^a^	21.55 ± 7.45 ^ab^
**10**	98.31 ± 14.45 ^a^	36.55 ± 2.27 ^b^	15.88 ± 3.00 ^b^	12. 97 ± 0.50 ^a^	21.57 ± 1.43 ^ab^
**11**	132.18 ± 21.12 ^bc^	36.61 ± 2.67 ^b^	13.73 ± 1.13 ^b^	15.82 ± 1.28 ^a^	13.83 ± 4.21 ^a^
**12**	133.07 ± 20.33 ^bc^	36.70 ± 2.55 ^b^	13.11 ± 1.15 ^b^	15.90 ± 1.04 ^a^	13.42 ± 3.66 ^a^
**Sig.**	**	**	**	**	**

^1^ Results are expressed as mg of gallic acid/100 g of fresh weight; ^2^ Results are expressed as mg of cyanidin-3-O-glucoside/100 g of fresh weight; ^3^ Results are expressed as mg of catechin/100 g of fresh weight; ^4^ Results are expressed as mg of ascorbic acid/100 g of fresh weight; ^5^ Results are expressed as mg of ascorbic acid/100 g of fresh weight.

**Table 8:** Fruits stored at –20 °C in freezer, packaged in PET film. Effect of storage period on the total phenolic content, total monomeric anthocyanin content, total flavonoid content, the ascorbic acid content, and the antioxidant activity DPPH parameters in red raspberries (cv. Erika). The results are the mean of three replicates ± Standard Deviation. ** (*P* ≤ 0.01). The letters represent the significant difference vertically at *P* ≤ 0.05.

Month	Total Phenolic Content ^1^	Total Monomeric Anthocyanin Content ^2^	Total Flavonoids Content ^3^	Ascorbic Acid Content ^4^	AA DPPH Assay ^5^
**1**	167.10 ± 4.69 ^bc^	35.98 ± 7.78 ^cd^	15.80 ± 1.41 ^ab^	28.81 ± 1.26 ^d^	81.31 ± 18.81 ^c^
**2**	169.45 ± 4.40 ^bc^	36.73 ± 5.95 ^cd^	18.80 ± 1.41 ^ab^	41.84 ± 5.86 ^e^	42.12 ± 15.27 ^c^
**3**	189.19 ± 14.67 ^c^	42.27 ± 7.05 ^d^	25.92 ± 1.76 ^b^	19.76 ± 1.33 ^bc^	55.21 ± 7.32 ^bc^
**4**	128.27 ± 14.10 ^ab^	38.88 ± 3.03 ^cd^	14.16 ± 1.10 ^ab^	38.13 ± 4.47 ^e^	87.81 ± 42.49 ^ab^
**5**	193.35 ± 35.30 ^c^	44.14 ± 6.06 ^d^	6.56 ± 1.17 ^a^	25.11 ± 2.61 ^cd^	26.31 ± 1.35 ^ab^
**6**	108.22 ± 3.77 ^a^	32.14 ± 5.85 ^bcd^	7.55 ± 1.21 ^a^	16.35 ± 1.13 ^ab^	29.51 ± 5.13 ^ab^
**7**	168.63 ± 33.80 ^bc^	21.68 ± 3.97 ^a^	9.48 ± 2.63 ^a^	18.72 ± 2.03 ^b^	28.71 ± 4.64 ^ab^
**8**	87.69 ± 20.29 ^a^	30.42 ± 2.86 ^abc^	13.77 ± 3.00 ^ab^	15.94 ± 0.11 ^ab^	39.75 ± 4.34 ^ab^
**9**	121.24 ± 27.88 ^a^	36.38 ± 2.71 ^cd^	12.24 ± 2.23 ^ab^	14.07 ± 0.44 ^ab^	19.22 ± 3.34 ^ab^
**10**	93.96 ± 15.95 ^a^	24.39 ± 4.05 ^ab^	14.11 ± 20.67 ^ab^	12.34 ± 0.48 ^a^	16.83 ± 3.37 ^a^
**11**	116.45 ± 22.48 ^a^	40.97 ± 0.89 ^cd^	10.19 ± 3.05 ^a^	18.90 ± 1.95 ^b^	31.82 ± 2.69 ^ab^
**12**	116.90 ± 20.07 ^a^	40.55 ± 0.95 ^cd^	10.88 ± 3.48 ^a^	19.21 ± 1.12 ^b^	31.97 ± 2.31 ^ab^
**Sig.**	**	**	**	**	**

^1^ Results are expressed as mg of gallic acid/100 g of fresh weight; ^2^ Results are expressed as mg of cyanidin-3-O- glucoside/100 g of fresh weight; ^3^ Results are expressed as mg of catechin/100 g of fresh weight; ^4^ Results are expressed as mg of ascorbic acid/100 g of fresh weight; ^5^ Results are expressed as mg of ascorbic acid/100 g of fresh weight.

**Table 9:** Fruits stored at –20 °C in freezer, without packaging film. Effect of storage period on the total phenolic content, total monomeric anthocyanin content, total flavonoid content, the ascorbic acid content, and the antioxidant activity DPPH assay parameters in red raspberries (cv. Erika). The results are the mean of three replicates ± Standard Deviation. ** (*P* ≤ 0.01); n.s. (not significant). The letters represent the significant difference vertically at *P* ≤ 0.05.

Month	Total Phenolic Content ^1^	Total Monomeric Anthocyanin Content ^2^	Total Flavonoids Content ^3^	Ascorbic Acid Content ^4^	AA DPPH Assay ^5^
**1**	177.33 ± 2.61 ^e^	34.60 ± 2.33 ^ab^	17.85 ± 0.90 ^d^	29.41 ± 2.66 ^e^	51.31 ± 11.67 ^bc^
**2**	174.45 ± 4.31 ^e^	35.98 ± 2.34 ^ab^	20.85 ± 0.90 ^e^	55.60 ± 12.89 ^g^	55.78 ± 6.42 ^c^
**3**	185.74 ± 21.81 ^e^	39.60 ± 8.49 ^ab^	24.15 ± 2.87 ^e^	26.70 ± 7.00 ^de^	38.34 ± 10.21 ^abc^
**4**	119.39 ± 0.44 ^bc^	37.99 ± 10.57 ^ab^	12.66 ± 0.31 ^c^	42.12 ± 6.12 ^f^	83.37 ± 13.47 ^d^
**5**	167.44 ± 28.49 ^de^	42.29 ± 3.19 ^b^	8.71 ± 3.78 ^b^	25.56 ± 1.80 ^cde^	29.80 ± 5.32 ^abc^
**6**	112.34 ± 55.79 ^cd^	27.78 ± 5.68 ^ab^	7.95 ± 3.42 ^b^	19.66 ± 1.69 ^abcd^	30.01 ± 5.07 ^abc^
**7**	77.11 ± 6.19 ^abc^	28.84 ± 7.93 ^ab^	3.52 ± 1.21 ^a^	22.82 ± 2.34 ^bcde^	23.10 ± 8.04 ^ab^
**8**	71.14 ± 18.44 ^ab^	36.59 ± 1.33 ^ab^	10.48 ± 3.63 ^c^	12.75 ± 1.25 ^ab^	29.09 ± 2.37 ^abc^
**9**	113.13 ± 15.21 ^bc^	39.50 ± 1.69 ^ab^	8.05 ± 0.82 ^b^	11.94 ± 1.78 ^ab^	29.70 ± 4.10 ^abc^
**10**	57.96 ± 12.08 ^a^	29.11 ± 5.32 ^ab^	11.80 ± 19.67 ^c^	10.83 ± 0.74 ^a^	11.30 ± 5.41 ^a^
**11**	96.59 ± 1.03 ^abc^	28.18 ± 6.42 ^a^	7.35 ± 1.22 ^b^	15.00 ± 1.84 ^abc^	34.81 ± 11.31 ^abc^
**12**	97.12 ± 1.45 ^abc^	27.66 ± 5.70 ^a^	7.22 ± 1.36 ^b^	15.76 ± 1.54 ^abc^	34.99 ± 7.44 ^abc^
**Sig.**	**	n.s.	**	**	**

^1^ Results are expressed as mg of gallic acid/100 g of fresh weight; ^2^ Results are expressed as mg of cyanidin-3-O-glucoside/100 g of fresh weight; ^3^ Results are expressed as mg of catechin/100 g of fresh weight; ^4^ Results are expressed as mg of ascorbic acid/100 g of fresh weight; ^5^ Results are expressed as mg of ascorbic acid/100 g of fresh weight.

**Table 10:** Variance analyses on the different parameters, taking into consideration film effect, day or month effect and the interaction. ** (*P* ≤ 0.01); * (*P* ≤ 0.05); n.s. (not significant).

Parameter	Refrigerated Fruits			Frozen Fruits		
	Film	Days	Film and Days	Film	Months	Film and Months
Total Phenolic content	**	**	n.s.	n.s.	n.s.	**
Total monomeric Anthocyanin content	**	**	**	n.s.	**	n.s.
Total Flavonoid content	n.s.	**	n.s.	n.s.	**	n.s.
Ascorbic acid content	n.s.	n.s.	**	n.s.	**	**
AA (DPPH assay)	n.s.	*	**	n.s.	**	n.s.
Cyanidin-3-O-sophoroside	**	**	*	n.s.	**	**
Cyanidin-3-O-Glucoside	**	**	n.s.	n.s.	**	*
Cyanidin-3-O-rutinoside	**	n.s.	**	n.s.	**	n.s.
Pelargonidin-3-O-Glucoside	**	n.s.	**	n.s.	**	**

**Table 11:** Effect of storage period, from the fruits at harvest on the different anthocyanidin compounds content in red raspberries (cv. Erika). Results are expressed in µg/g of fresh weight. The results are the mean of three replicates ± Standard Deviation.

Day	Cyanidin-3-O-sophoroside	Cyanidin-3-O-glucoside	Cyanidin-3-O-rutinoside	Pelargonidin-3-O-glucoside
**0**	73.49 ± 1.02	55.84 ± 0.95	66.67 ± 2.33	20.46 ± 1.49

**Table 12:** Fruits stored at –20 °C in freezer, packaged in Nanoactive A film. Effect of storage period on the different anthocyanidin compounds content in red raspberries (cv. Erika). Results are expressed in µg/g of fresh weight. The results are the mean of three replicates ± Standard Deviation. ** (*P* ≤ 0.01); * (*P* ≤ 0.05); n.s. (not significant). The letters represent the significant difference vertically at *P* ≤ 0.05.

Month	Cyanidin-3-O-sophoroside	Cyanidin-3-O-glucoside	Cyanidin-3-O-rutinoside	Pelargonidin-3-O-glucoside
**1**	69.41 ± 0.04 ^de^	55.01 ± 1.45 ^f^	67.05 ± 1.85 ^a^	21.58 ± 1.48 ^de^
**2**	69.38 ± 0.88 ^de^	54.12 ± 1.34 ^ef^	65.84 ± 2.66 ^a^	22.72 ± 0.71 ^e^
**3**	62.83 ± 0.82 ^c^	48.55 ± 1.55 ^bcde^	55.85 ± 0.09 ^a^	20.85 ± 0.38 ^cde^
**4**	53.35 ± 3.28 ^ab^	47.85 ± 3.75 ^bcd^	50.36 ± 6.02 ^a^	15.77 ± 2.56 ^a^
**5**	68.68 ± 0.91 ^cde^	51.17 ± 0.55 ^cdef^	61.12 ± 4.75 ^a^	20.66 ± 1.46 ^cde^
**6**	69.51 ± 0.37 ^de^	53.00 ± 0.53 ^def^	60.39 ± 1.62 ^a^	20.35 ± 0.57 ^bcde^
**7**	72.03 ± 1.78 ^e^	52.69 ± 0.38 ^def^	68.20 ± 7.81 ^a^	20.30 ± 1.29 ^bcde^
**8**	52.48 ± 4.07 ^ab^	46.24 ± 3.73 ^bc^	64.05 ± 15.89 ^a^	17.20 ± 1.00 ^abc^
**9**	55.52 ± 0.30 ^b^	43.47 ± 0.03 ^b^	62.48 ± 0.36 ^a^	18.25 ± 0.15 ^abcd^
**10**	48.25 ± 0.22 ^a^	37.32 ± 0.65 ^a^	49.42 ± 1.06 ^a^	16.68 ± 0.12 ^ab^
**11**	65.08 ± 1.73 ^cd^	49.61 ± 0.63 ^cdef^	67.12 ± 2.45 ^a^	21.68 ± 0.09 ^de^
**12**	65.33 ± 1.26 ^cd^	49.78 ± 0.79 ^cdef^	67.88 ± 2.22 ^a^	22.22 ± 0.11 ^de^
**Sig.**	**	**	n.s.	*

**Table 13:** Fruits stored at –20 °C in freezer without packaging film. Effect of storage period on the different anthocyanidin compounds content in red raspberries (cv. Erika). Results are expressed in µg/g of fresh weight. The results are the mean of three replicates ± Standard Deviation. ** (*P* ≤ 0.01); n.s. (not significant). The letters represent the significant difference vertically at *P* ≤ 0.05.

Month	Cyanidin-3-O-sophoroside	Cyanidin-3-O-glucoside	Cyanidin-3-O-rutinoside	Pelargonidin-3-O-glucoside
**1**	69.79 ± 2.16 ^bc^	55.28 ± 1.12 ^bc^	64.96 ± 2.07 ^ab^	23.99 ± 0.28 ^d^
**2**	70.74 ± 1.51 ^bc^	54.32 ± 1.25 ^bc^	69.16 ± 1.56 ^b^	21.44 ± 0.84 ^bcd^
**3**	62.53 ± 0.69 ^bc^	46.38 ± 0.20 ^b^	54.86 ± 1.38 ^a^	19.55 ± 0.82 ^bc^
**4**	64.18 ± 1.39 ^bc^	46.54 ± 0.66 ^b^	60.96 ± 4.58 ^ab^	20.31 ± 0.28 ^bc^
**5**	73.97 ± 2.88 ^c^	56.82 ± 0.92 ^c^	61.96 ± 2.15 ^ab^	22.36 ± 0.82 ^cd^
**6**	68.62 ± 0.10 ^bc^	55.16 ± 1.15 ^bc^	70.85 ± 3.41 ^b^	21.23 ± 0.83 ^bcd^
**7**	72.23 ± 10.51 ^bc^	53.81 ± 7.78 ^bc^	63.78 ± 7.51 ^ab^	14.67 ± 1.48 ^a^
**8**	70.82 ± 3.96 ^bc^	52.91 ± 3.26 ^bc^	60.26 ± 3.85 ^ab^	20.15 ± 1.66 ^bc^
**9**	72.59 ± 0.07 ^bc^	56.00 ± 1.55 ^c^	60.45 ± 0.40 ^ab^	21.96 ± 0.53 ^bcd^
**10**	41.61 ± 0.72 ^a^	34.38 ± 0.42 ^a^	53.46 ± 1.34 ^a^	13.06 ± 0.25 ^a^
**11**	61.22 ± 0.80 ^b^	49.08 ± 0.85 ^bc^	67.90 ± 1.39 ^b^	19.18 ± 0.76 ^b^
**12**	61.70 ± 0.66 ^b^	49.76 ± 0.99 ^bc^	68.21 ± 1.04 ^b^	19.84 ± 0.50 ^b^
**Sig.**	**	**	n.s.	**

**Table 14:** Fruits stored at 1 °C in fridge, packaged in Nanoactive A film. Effect of storage period on the different anthocyanidin compounds content in red raspberries (cv. Erika). Results are expressed in µg/g of fresh weight. The results are the mean of three replicates ± Standard Deviation. ** (*P* ≤ 0.01); * (*P* ≤ 0.05); n.s. (not significant). The letters represent the significant difference vertically at *P* ≤ 0.05.

Day	Cyanidin-3-O-sophoroside	Cyanidin-3-O-glucoside	Cyanidin-3-O-rutinoside	Pelargonidin-3-O-glucoside
**1**	73.82 ± 2.92 ^cd^	56.07 ± 1.47 ^a^	61.12 ± 5.91 ^abc^	22.03 ± 0.43 ^cde^
**2**	65.71 ± 0.83 ^abc^	52.90 ± 1.44 ^a^	63.05 ± 5.45 ^abcd^	20.88 ± 0.39 ^bcd^
**3**	69.47 ± 1.42 ^bcd^	53.27 ± 0.64 ^a^	71.99 ± 0.35 ^abcd^	22.58 ± 0.14 ^de^
**4**	69.20 ± 5.01 ^bcd^	52.30 ± 3.55 ^a^	63.45 ± 3.32 ^abcd^	21.70 ± 1.39 ^cde^
**5**	72.51 ± 0.11 ^bcd^	53.28 ± 0.11 ^a^	64.17 ± 0.92 ^abcd^	22.03 ± 0.23 ^cde^
**6**	74.69 ± 2.92 ^d^	54.32 ± 0.79 ^a^	67.45 ± 3.11 ^bcd^	22.41 ± 0.20 ^de^
**7**	70.28 ± 0.98 ^bcd^	54.33 ± 0.07 ^a^	65.73 ± 1.49 ^abcd^	20.90 ± 0.84 ^bcd^
**8**	71.70 ± 1.29 ^bcd^	56.38 ± 1.84 ^a^	66.67 ± 0.20 ^abcd^	25.26 ± 0.76 ^f^
**9**	63.80 ± 3.88 ^ab^	50.60 ± 1.86 ^a^	60.25 ± 3.14 ^ab^	21.19 ± 0.09 ^bcd^
**10**	68.16 ± 1.71 ^bcd^	53.67 ± 0.27 ^a^	63.76 ± 1.71 ^abcd^	19.99 ± 0.40 ^abc^
**11**	67.63 ± 0.07 ^abcd^	55.36 ± 2.21 ^a^	70.10 ± 0.32 ^abcd^	19.35 ± 0.30 ^ab^
**12**	59.53 ± 1.51 ^a^	50.16 ± 1.60 ^a^	57.05 ± 0.78 ^a^	18.69 ± 0.25 ^a^
**13**	72.93 ± 1.52 ^cd^	55.44 ± 1.69 ^a^	62.81 ± 0.50 ^abcd^	23.32 ± 0.07 ^e^
**14**	71.99 ± 4.50 ^bcd^	54.97 ± 3.50 ^a^	60.42 ± 2.74 ^abc^	19.99 ± 1.02 ^abc^
**Sig.**	*	n.s.	n.s.	**

**Table 15:** Fruits stored at 1 °C in fridge without packaging. Effect of storage period on the different anthocyanidin compounds content in red raspberries (cv. Erika). Results are expressed in µg/g of fresh weight. The results are the mean of three replicates ± Standard Deviation. n.s. (not significant). The letters represent the significant difference vertically at *P* ≤ 0.05.

Day	Cyanidin-3-O-sophoroside	Cyanidin-3-O-glucoside	Cyanidin-3-O-rutinoside	Pelargonidin-3-O-glucoside
**1**	81.42 ± 4.35 ^d^	55.08 ± 0.70 ^c^	60.36 ± 6.33 ^a^	20.33 ± 0.73 ^ab^
**2**	69.21 ± 3.30 ^ab^	50.65 ± 2.36 ^abc^	59.42 ± 3.30 ^a^	21.50 ± 1.85 ^b^
**3**	75.21 ± 0.56 ^abc^	53.04 ± 0.48 ^bc^	61.72 ± 2.43 ^a^	20.53 ± 0.53 ^ab^
**4**	70.57 ± 1.96 ^abc^	46.81 ± 2.93 ^ab^	56.04 ± 1.30 ^a^	19.40 ± 1.54 ^ab^
**5**	80.05 ± 0.90 ^cd^	52.40 ± 0.47 ^bc^	60.62 ± 0.49 ^a^	19.85 ± 0.41 ^ab^
**6**	76.08 ± 0.79 ^cd^	48.51 ± 0.44 ^abc^	56.04 ± 0.64 ^a^	17.87 ± 0.53 ^ab^
**7**	64.51 ± 6.00 ^a^	43.77 ± 4.31 ^a^	52.36 ± 3.79 ^a^	17.36 ± 1.53 ^a^
**8**	79.15 ± 0.69 ^cd^	53.91 ± 0.63 ^bc^	59.73 ± 1.17 ^a^	20.58 ± 0.02 ^ab^
**9**	73.42 ± 5.02 ^abc^	49.19 ± 3.61 ^abc^	57.61 ± 4.18 ^a^	19.26 ± 1.60 ^ab^
**10**	78.96 ± 2.66 ^cd^	51.00 ± 1.31 ^abc^	60.31 ± 0.39 ^a^	19.62 ± 0.14 ^ab^
**Sig.**	n.s.	n.s.	n.s.	n.s.

**Table 16:** Fruits stored at 1 °C in fridge, packaged in PET film. Effect of storage period on the different anthocyanidin compounds content in red raspberries (cv. Erika). Results are expressed in µg/g of fresh weight. The results are the mean of three replicates ± Standard Deviation. ** (*P* ≤ 0.01); * (*P* ≤ 0.05); n.s. (not significant). The letters represent the significant difference vertically at *P* ≤ 0.05.

Day	Cyanidin-3-O-sophoroside	Cyanidin-3-O-glucoside	Cyanidin-3-O-rutinoside	Pelargonidin-3-O-glucoside
**1**	73.82 ± 0.05 ^cde^	54.60 ± 1.97 ^c^	66.38 ± 0.25 ^e^	21.30 ± 0.27 ^abc^
**2**	61.55 ± 2.81 ^ab^	51.10 ± 0.13 ^bc^	57.00 ± 0.72 ^bc^	21.44 ± 2.40 ^bc^
**3**	71.38 ± 3.08 ^cde^	53.76 ± 1.89 ^bc^	63.14 ± 0.64 ^de^	20.65 ± 0.04 ^abc^
**4**	68.36 ± 3.36 ^abcde^	50.79 ± 2.25 ^bc^	59.41 ± 2.29 ^bcd^	20.75 ± 0.64 ^abc^
**5**	76.36 ± 1.90 ^e^	54.65 ± 0.31 ^c^	65.67 ± 0.03 ^e^	20.54 ± 0.61 ^abc^
**6**	75.13 ± 0.47 ^de^	51.66 ± 0.66 ^bc^	61.45 ± 0.46 ^cde^	19.97 ± 1.17 ^abc^
**7**	65.63 ± 4.56 ^abcd^	47.68 ± 2.72 ^ab^	56.28 ± 0.96 ^bc^	18.49 ± 0.84 ^abc^
**8**	74.47 ± 1.51 ^cde^	50.71 ± 2.55 ^bc^	59.85 ± 2.55 ^bcd^	18.62 ± 1.08 ^abc^
**9**	60.59 ± 1.56 ^a^	42.07 ± 2.33 ^a^	49.44 ± 2.60 ^a^	17.76 ± 1.16 ^ab^
**10**	70.65 ± 1.78 ^bcde^	49.64 ± 0.85 ^bc^	63.69 ± 0.85 ^de^	21.90 ± 0.91 ^c^
**11**	77.99 ± 3.08 ^e^	51.47 ± 2.93 ^bc^	58.87 ± 2.24 ^bcd^	17.92 ± 1.39 ^ab^
**12**	64.95 ± 5.12 ^abc^	47.59 ± 1.20 ^ab^	54.70 ± 1.92 ^b^	17.51 ± 0.61 ^a^
**Sig.**	**	*	**	n.s.

**Table 17:** Fruits stored at –20 °C in freezer, packaged in Nanoactive B film. Effect of storage period on the different anthocyanidin compounds content in red raspberries (cv. Erika). Results are expressed in µg/g of fresh weight. The results are the mean of three replicates ± Standard Deviation. ** (*P* ≤ 0.01). The letters represent the significant difference vertically at *P* ≤ 0.05.

Month	Cyanidin-3-O-sophoroside	Cyanidin-3-O-glucoside	Cyanidin-3-O-rutinoside	Pelargonidin-3-O-glucoside
**1**	69.91 ± 0.20 ^c^	53.46 ± 0.22 ^cd^	71.11 ± 0.13 ^cde^	21.66 ± 0.72 ^bcd^
**2**	65.43 ± 0.35 ^bc^	58.20 ± 0.26 ^cd^	75.70 ± 0.20 ^e^	18.94 ± 0.51 ^b^
**3**	72.39 ± 1.00 ^c^	52.31 ± 1.26 ^bcd^	59.73 ± 3.36 ^bc^	23.56 ± 0.78 ^cd^
**4**	72.13 ± 7.73 ^c^	52.40 ± 5.09 ^bcd^	54.94 ± 7.90 ^b^	21.35 ± 1.87 ^bcd^
**5**	70.32 ± 2.01 ^c^	54.18 ± 1.93 ^cd^	73.45 ± 3.58 ^de^	22.34 ± 0.13 ^bcd^
**6**	74.25 ± 2.72 ^c^	61.58 ± 1.60 ^d^	60.49 ± 0.36 ^bc^	24.66 ± 0.22 ^d^
**7**	66.79 ± 8.50 ^bc^	51.56 ± 6.34 ^bc^	62.15 ± 7.48 ^bcd^	20.30 ± 2.69 ^bc^
**8**	55.87 ± 2.71 ^b^	42.99 ± 1.55 ^b^	57.67 ± 2.48 ^b^	19.03 ± 1.07 ^b^
**9**	67.53 ± 0.48 ^bc^	51.07 ± 0.60 ^bc^	70.87 ± 1.33 ^cde^	23.68 ± 0.46 ^cd^
**10**	42.09 ± 3.00 ^a^	33.07 ± 2.48 ^a^	42.24 ± 2.70 ^a^	13.08 ± 1.49 ^a^
**11**	66.57 ± 3.47 ^bc^	52.16 ± 0.98 ^bcd^	75.37 ± 1.29 ^e^	21.94 ± 0.16 ^bcd^
**12**	66.83 ± 2.25 ^bc^	52.72 ± 0.65 ^bcd^	76.13 ± 1.15 ^e^	22.14 ± 0.23 ^bcd^
**Sig.**	**	**	**	**

**Table 18:** Fruits stored at –20 °C in freezer, packaged in PET film. Effect of storage period on the different anthocyanidin compounds content in red raspberries (cv. Erika). Results are expressed in µg/g of fresh weight. The results are the mean of three replicates ± Standard Deviation. ** (*P* ≤ 0.01). The letters represent the significant difference vertically at *P* ≤ 0.05.

Month	Cyanidin-3-O-sophoroside	Cyanidin-3-O-glucoside	Cyanidin-3-O-rutinoside	Pelargonidin-3-O-glucoside
**1**	63.87 ± 1.65 ^bcd^	48.56 ± 0.28 ^b^	59.61 ± 0.50 ^bc^	19.79 ± 0.45 ^b^
**2**	66.88 ± 0.31 ^bcde^	53.70 ± 0.72 ^bc^	70.74 ± 3.71 ^de^	22.24 ± 0.19 ^bc^
**3**	68.07 ± 3.50 ^bcde^	51.00 ± 2.70 ^bc^	51.90 ± 5.90 ^ab^	22.73 ± 1.94 ^bc^
**4**	75.11 ± 6.03 ^e^	55.25 ± 1.20 ^c^	64.39 ± 2.12 ^cde^	22.40 ± 0.52 ^bc^
**5**	73.75 ± 3.16 ^de^	55.73 ± 2.02 ^c^	63.02 ± 4.97 ^cde^	21.98 ± 1.67 ^bc^
**6**	70.74 ± 0.82 ^cde^	55.57 ± 0.94 ^c^	72.95 ± 0.82 ^e^	22.13 ± 1.22 ^bc^
**7**	67.36 ± 1.55 ^bcde^	52.77 ± 1.19 ^bc^	67.27 ± 0.70 ^cde^	20.00 ± 0.13 ^b^
**8**	73.88 ± 0.49 ^e^	52.80 ± 1.08 ^bc^	62.96 ± 1.84 ^cde^	19.84 ± 0.19 ^b^
**9**	63.61 ± 5.11 ^bc^	48.79 ± 3.30 ^b^	48.77 ± 4.07 ^a^	25.73 ± 2.23 ^c^
**10**	44.39 ± 0.11 ^a^	34.41 ± 0.05 ^a^	45.43 ± 0.02 ^a^	13.11 ± 0.00 ^a^
**11**	60.59 ± 1.11 ^b^	54.05 ± 0.66 ^bc^	61.69 ± 0.06 ^cd^	19.49 ± 0.04 ^b^
**12**	60.91 ± 1.03 ^b^	54.79 ± 0.50 ^bc^	62.03 ± 0.10 ^cd^	19.84 ± 0.08 ^b^
**Sig.**	**	**	**	**

**Table 19:** Fruits stored at 1 °C in fridge, packaged in Nanoactive B film. Effect of storage period on the different anthocyanidin compounds content in red raspberries (cv. Erika). Results are expressed in µg/g of fresh weight. The results are the mean of three replicates ± Standard Deviation. n.s. (not significant). The letters represent the significant difference vertically at *P* ≤ 0.05.

ay	Cyanidin-3-O-sophoroside	Cyanidin-3-O-glucoside	Cyanidin-3-O-rutinoside	Pelargonidin-3-O-glucoside
**1**	71.04 ± 1.65 ^a^	55.27 ± 0.01 ^a^	68.05 ± 3.43 ^ab^	22.02 ± 0.31 ^ab^
**2**	68.48 ± 3.56 ^a^	51.18 ± 4.62 ^a^	63.94 ± 6.72 ^ab^	19.58 ± 2.13 ^ab^
**3**	68.87 ± 1.13 ^a^	58.42 ± 5.69 ^a^	56.42 ± 4.15 ^a^	18.33 ± 1.11 ^a^
**4**	66.39 ± 0.12 ^a^	53.82 ± 1.03 ^a^	72.21 ± 0.81 ^b^	19.97 ± 0.65 ^ab^
**5**	65.27 ± 0.54 ^a^	54.15 ± 0.07 ^a^	72.64 ± 0.47 ^b^	21.90 ± 0.51 ^ab^
**6**	66.59 ± 0.23 ^a^	51.48 ± 0.24 ^a^	66.99 ± 1.46 ^ab^	23.33 ± 1.12 ^b^
**7**	67.43 ± 1.30 ^a^	55.63 ± 0.49 ^a^	66.47 ± 1.94 ^ab^	23.44 ± 0.97 ^b^
**Sig.**	n.s.	n.s.	n.s.	n.s.

## Supplementary Materials

The following are available online at https://www.mdpi.com/2076-3921/8/8/254/s1.
