# Supplementary material for: The Influence of Film and Storage on the Phenolic and Antioxidant Properties of Red Raspberries (Rubus idaeus L.) cv. Erika
Source: Antioxidants (Basel). 2019 Jul 30;8(8):254. doi: 10.3390/antiox8080254 (PMC6719043; doi:10.3390/antiox8080254)
Supplement: Supplementary file 1 [file antioxidants-08-00254-s001.pdf]

Determination of Total Phenolic Content (TPC)

Preparation of the blank. One mL of Folin-Ciocalteu was mixed with 5 mL of deionised water. After 8 minutes, 10 mL of sodium carbonate was added, then it was brought to volume with deionised water to 25 mL. The calibration curve was made with gallic acid in these concentrations: 0.8, 2.0, 4.0, 8.0 and 10.0 mg/L. The concentration of the sample was expressed in mg/100g of fruit, and is given by the following formula:

$$((C \times DF \times mg) / g) \times 100$$

Where: **C** is the concentration of the sample; **DF** is the dilution factor of 25; **mg** indicates milligrams of the initial sample and **g** are the grams of the fruit used.

Determination of total monomeric anthocyanin content (TMAC).

Preparation of buffer solutions. (a) Buffer pH 1.0 with potassium chloride, 0.025 M. Obtained by weighing 1.86 g of KCl in a beaker and adding 980 mL of deionised water. The pH was measured and was stabilised at pH 1.0 ± 0.05 with HCl (about 6.3 mL) and made up to volume in a 1 L volumetric flask with deionised water. (b) Buffer pH 4.5 with 0.4 M sodium acetate Obtained by weighing 54.43 g of $CH_3CO_2Na \bullet 3H_2O$ in a beaker and adding 960 mL of deionised water. The pH was measured and regularised at pH 4.5 ± 0.05 with HCl (about 20 mL) and made up to volume in a volumetric flask with deionised water 1L.
